# Novel model to predict survival in DCD heart transplants: Development of a mortality risk score using UNOS data

**DOI:** 10.1016/j.jhlto.2026.100530

**Published:** 2026-03-03

**Authors:** Anh Nguyen, Abbas Rana, Alexis Shafii, Gabriel Loor, Andrew Civitello, Todd Rosengart, Kenneth Liao

**Affiliations:** a Division of Cardiothoracic Transplantation and Circulatory Support, Department of Surgery, Baylor College of Medicine, Houston, TX; b Division of Abdominal Transplantation, Department of Surgery, Baylor College of Medicine, Houston, TX; c Division of Cardiology, Department of Medicine, Baylor College of Medicine, Houston, TX; d Division of Cardiothoracic Surgery, Department of Surgery, Baylor College of Medicine, Houston, TX

**Keywords:** Heart transplant, Survival, Mortality, Risk score, Donation after circulatory death

## Abstract

**Background:**

While prior studies have compared outcomes between donation after brain death (DBD) and donation after circulatory death (DCD) heart transplants, this study focuses on donor and recipient characteristics influencing survival and develop a risk score exclusively in DCD heart transplants.

**Methods:**

We analyzed adult DCD heart transplants in the UNOS database from January 2019 to June 2024. Mortality risk factors were assessed using multivariable Cox proportional hazards regression. The coefficients of the most significant factors were used to develop a mortality risk score.

**Results:**

Among 18,014 adult heart transplants, 1453 (8.1%) were DCD recipients. One-, two-, and three-year survival rates were 91.6%, 84.8%, and 80.3%, respectively. Statistically significant factors associated with increased mortality included recipient age >65 (HR=1.62, p=0.025), recipient female sex (HR=1.87, p=0.007), recipient diabetes (HR=1.64, p=0.009), prior cardiac surgery (HR=1.46, p=0.049), donor male sex (HR=1.96, p=0.034), longer ischemic time (HR=1.11 per hour, p=0.014) and CMV mismatch: donor-/recipient+ (HR=2.07, p=0.015); donor+/recipient- (HR=1.84, p=0.041). Though not statistically significant (p>0.05), donor age≥45, donor diabetes, donor cause of death, lung retrieval, recipients’ pretransplant dialysis, IV inotropes and mechanical ventilation were also added to the mortality risk score model due to its large effect sizes and clinical relevance.

**Conclusions:**

We developed a mortality risk score for DCD heart transplants including 7 recipient factors: age >65, female sex, diabetes, prior cardiac surgery, pretransplant ventilator use, IV inotropes; 7 donor factors: age≥45, male sex, diabetes, ischemic time>4 h, cause of death, lung retrieval and CMV positive in either donors or recipients.

## Introduction

Heart failure (HF) remains a major global health issue, impacting over 64 million individuals worldwide and more than 6.7 million adults in the United States each year.^1^ Heart transplantation is the most effective treatment for advanced HF, offering substantial improvements in both survival and quality of life. However, the growing demand for donor hearts far exceeds the available supply.^2^ Alarmingly, over 30% of patients on the transplant waiting list die within a year if they do not receive a heart.^3^ While traditional donation after brain death (DBD) is the primary source of donor hearts, incorporating donation after cardiac death (DCD) could help alleviate the shortage. Estimates suggest that using DCD hearts could expand the donor pool by up to 30%.^4^ Despite this potential, DCD heart transplants are less commonly performed due to concerns about warm ischemic damage and increased risk of cardiac complications during surgery.^5^, ^6^ Since its implementation in the US in 2019, there has been a significant number of studies comparing survival rates between DBD and DCD heart transplants.7., 8, 9, 10, 11, 12, 13, 14 However, the focus of our study is solely on the DCD heart transplants, aiming to identify donor and recipient risk factors associated with increased mortality in this population. Several heart transplant mortality risk scores have been developed based on data from the United Network for Organ Sharing (UNOS)^15^, ^16^, ^17^, ^18^, ^19^, ^20^, ^21^, ^22^ They are based on UNOS data prior to 2018, before the adoption of DCD heart transplantation in the U.S. The mortality risk factors identified for DBD heart transplants might not necessarily be the same for DCD ones. To address this gap, we utilize UNOS data from 2019 to 2024 to develop a risk score specifically tailored to the DCD heart transplant population.

## Methods

### Data source

We conducted a retrospective cohort study of adult heart transplant cases between January 2019 and June 2024, using data from the UNOS Standard Transplant Analysis and Research dataset. From the thoracic organ transplant recipient records, we identified 1453 adult heart transplants performed using DCD donors, after excluding cases involving DBD donors and combined heart-lung transplants.

### Statistical analysis

Donor and recipient characteristics were reported as frequencies and proportions for categorical variables and as median and interquartile range (IQR) for continuous variables. Multivariable Cox proportional hazards regression was used to identify risk factors associated with mortality. A total of 141 deaths were observed and included as events in the Cox proportional hazards model. The median follow‑up time was 11 months (IQR 3–18), and missing data were handled using a complete‑case approach without imputation. We evaluated the proportional hazards (PH) assumption for the Cox regression model using Schoenfeld residual–based tests. The global test indicated no violation of the PH assumption (p = 0.7672), and no individual covariate demonstrated statistically significant deviation from proportionality (all p-values> 0.05). Statistically significant and clinically relevant variables with large effect sizes were kept optimizing the model performance.23., 24 Model performance was evaluated using both discrimination and calibration metrics. Discrimination was assessed using the concordance (C‑) statistic with optimism-correction from bootstrap validation, and calibration was assessed using the Grønnesby–Borgan goodness‑of‑fit test based on deciles of predicted risk. To develop a clinically friendly risk score, we ran a Cox regression model with the 14 most significant variables and converted their beta-coefficients in the model to integer risk scores according to a well-established methodology.^25^ The total risk score of each patient was calculated based on these 14 risk factors and then classified as low (<25% percentile), moderate (25–75% percentile) and high risk (>75% percentile). Kaplan-Meier graphs were used to present survival by these three risk categories and the log-rank test to compare overall survival. All tests were 2 tailed with alpha level of 0.05. Analyses were performed on Stata version 18.0 (Stata Corp LLC, College Station, TX, USA).

This study was approved with a waiver of patient consent and HIPAA authorization by the Institutional Review Board for Human Subject Research for Baylor College of Medicine and Affiliated Hospitals (H-51123, approved on November 29, 2022).

## Results

### Recipient characteristics

Of the 18,014 heart transplants performed during the study period, 1453 (8.1%) utilized DCD hearts. Among these DCD recipients, the median age was 57 years (IQR 46–64), with a predominance of male patients (79.8%). The cohort was racially diverse, comprising 64.3% White, 22.9% Black, and 9.8% Hispanic individuals. Common comorbidities included obesity (36.2%) and diabetes (32.1%). A substantial proportion had undergone prior cardiac surgery (41.8%) or received a ventricular assist device (VAD) prior to transplant (34.1%). Most recipients were listed as UNOS status 1–3 (58.6%), reflecting higher clinical urgency, while 41.4% were status 4–6. DCD transplant volume increased steadily over time, with the majority (79.1%) performed at high-volume centers, representing the top third of institutions by DCD transplant activity. Detailed recipient characteristics are presented in Table 1.

**Table 1:** Baseline recipient characteristics in DCD heart transplants.

Age (years), median (Interquartile range)	57 (46-64)
Female, N (%)	294 (20.2)
Ethnicity, N (%)	
White Black Hispanic Asian American Indian Pacific Islander Multiracial	931 (64.3) 332 (22.9) 142 (9.8) 31 (2.1) 6 (0.4) 4 (0.3) 1 (0.1)
Obesity, N (%)	526 (36.2)
Diabetes, N (%)	466 (32.1)
Pretransplant dialysis, N (%)	63 (4.4)
Pretransplant cardiac surgery	607 (41.8)
Pretransplant inotropic support, N (%)	482 (33.2)
Pretransplant VAD, N (%)	495 (34.1)
Pretransplant ventilator, N (%)	13 (0.9)
Pretransplant ECMO, N (%)	38 (2.6)
Pretransplant IABP, N (%)	208 (14.3)
CMV positive, N (%)	759 (52.2)
End listing status, N (%)	
1 2 3 4 5 6	59 (4.1) 556 (38.3) 250 (17.2) 387 (26.6) 30 (2.1) 171 (11.8)
Transplant time	
2019 2020 2021 2022 2023 2024 (Jan-Jun)	7 105 196 330 585 230
Center volume ranking for DCD heart transplantation, N(%)	
Low Middle High	58 (4.0) 246 (16.9) 1,149 (79.1)

ECMO: Extracorporeal Membrane Oxygenation

IABP: Intra-Aortic Balloon Pump

VAD: Ventricular Assist Device

### Donor characteristics

DCD donors had a median age of 31 years (IQR 24–38), and the majority were male (83.7%). Most donors were White (76.3%), followed by Hispanic (11.9%) and Black (9.5%). A minority had comorbid conditions such as hypertension (13.2%) or diabetes (3.3%). Only 20% of donors had lower weights than 80% of the recipients’ weights. The median left ventricular ejection fraction was 62% (IQR 59–66). The median ischemic time for donor hearts was 5.1 h (IQR 3.5–6.5). CMV mismatch occurred in 49.2% of the cases. Donor causes of death were mostly anoxia (50.6%) and head trauma (41.8%). Lung retrieval was conducted in 23% of donors. Details are presented in Table 2.

**Table 2:** Baseline donor characteristics in DCD heart transplants.

Age (years), median (Interquartile range)	31 (24-38)
Male, N (%)	1,216 (83.7)
Ethnicity, N (%)	
White Black Hispanic Asian American Indian Pacific Islander Multiracial	1,105 (76.3) 137 (9.5) 172 (11.9) 17 (1.2) 12 (0.8) 0 (0.0) 6 (0.4)
Donor/Recipient weight < 80%, N (%)	290 (20.0)
Hypertension, N (%)	190 (13.2)
Diabetes, N (%)	47 (3.3)
Left ventricular ejection fraction, N (%)	62 (59-66)
Ischemic time (hours), median (IQR)	5.1 (3.5-6.5)
CMV status, N (%)	
Donor (-) / Recipient (-) Donor (-) / Recipient (+) Donor (+) / Recipient (+) Donor (+) / Recipient (-)	289 (20.5) 326 (23.1) 425 (30.2) 369 (26.1)
Donor cause of death, N (%)	
Anoxia Stroke Head trauma	708 (50.6) 106 (7.6) 584 (41.8)
Lung retrieval, N(%)	334 (23.0)

### Predictors of DCD heart transplant mortality

Multivariable Cox regression analysis identified several donor and recipient characteristics significantly associated with post-transplant mortality in DCD heart recipients. Among donor factors, male sex (HR=1.96, p=0.034) and CMV mismatch—specifically donor-/recipient+ (HR=2.07, p=0.015) and donor+/recipient- (HR=1.84, p=0.041)—were independently associated with increased mortality risk. Additionally, each hour of prolonged ischemic time was linked to a higher hazard of death (HR=1.11, p=0.014). On the recipient side, age greater than 65 years (HR=1.62, p=0.025), female sex (HR=1.87, p=0.007), diabetes (HR=1.64, p=0.009), and prior cardiac surgery (HR=1.46, p=0.049) were significant predictors of reduced survival. Although not statistically significant (p>0.05), donor age ≥45, donor diabetes, donor stroke, donor head trauma, lung retrieval and recipient pre-transplant dialysis, IV inotropes and ventilator use showed trends toward increased mortality with HRs of 1.27, 2.05, 1.26, 1.28, 1.24, 1.39, 1.24 and 2.71, respectively. Given their large effect sizes and clinical relevance, these variables were still kept in the risk score model together with other statistically significant ones. The final model demonstrated good discriminatory ability, with an optimism-corrected C-statistic of 0.71 (95% CI 0.67- 0.75) and acceptable calibration, as indicated by a non‑significant Grønnesby–Borgan test (p=0.103). Full details are presented in Table 3.

**Table 3:** Multivariable Survival Analysis for Post-DCD Heart Transplantation

Risk Factors	Hazard Ratio [95% CI]	P-value
Donor factors		

Donor age>=45	1.27 [0.53-3.04]	0.586
Male	1.96 [1.05-3.65]	0.034
CMV		
Donor (-) / Recipient (-)	Ref	
Donor (-) / Recipient (+)	2.07 [1.15-3.72]	0.015
Donor (+) / Recipient (+)	1.57 [0.87-2.85]	0.138
Donor (+) / Recipient (-)	1.84 [1.02-3.29]	0.041
Donor/Recipient Weight Ratio <0.8	1.28 [0.82-2.01]	0.289
Donor hypertension	0.75 [0.41-1.41]	0.375
Donor diabetes	2.05 [0.86-4.89]	0.107
Donor LVEF	0.98 [0.96-1.01]	0.193
Ischemic time (per hour)	1.11 [1.02-1.20]	0.014
Donor cause of death		
Anoxia	Ref	
Stroke Head trauma	1.26 [0.59-2.66] 1.28 [0.88-1.85]	0.554 0.198
Lung retrieval	1.24 [0.82-1.87]	0.310

Recipient factors		

Age>65	1.62 [1.06-2.48]	0.025
Female	1.87 [1.19-2.96]	0.007
Black	1.21 [0.80-1.83]	0.356
Obesity	0.92 [0.62-1.38]	0.692
Diabetes	1.64 [1.13-2.37]	0.009
Pre-transplant dialysis	1.39 [0.60-3.21]	0.439
Pre-transplant cardiac surgery	1.46 [1.00-2.14]	0.050
Pre-transplant IV inotropes	1.24 [0.81-1.92]	0.321
Pre-transplant VAD	1.09 [0.71-1.70]	0.688
Pre-transplant on ventilator	2.71 [0.53-14.02]	0.233
Pre-transplant ECMO	0.77 [0.15-4.04]	0.754
Pre-transplant IABP	0.91 [0.48-1.72]	0.777
End status		
1	Ref	
2	0.72 [0.19-2.80]	0.635
3	0.73 [0.18-2.94]	0.663
4	0.69 [0.17-2.74]	0.595
5	1.25 [0.22-7.13]	0.799
6	0.63 [0.14-2.78]	0.545
Transplant year		
2019	Ref	
2020	0.37 [0.10-1.39]	0.141
2021	0.57 [0.15-2.21]	0.417
2022	0.70 [0.18-2.75]	0.613
2023	0.86 [0.22-3.38]	0.824
2024	1.01 [0.21-4.75]	0.994
Center volume ranking for DCD heart transplantation		
Low	Ref	
Middle High	0.67 [0.23-1.92] 0.48 [0.18-1.26]	0.457 0.136

### Risk score of DCD heart transplant mortality

**Figure 1:**
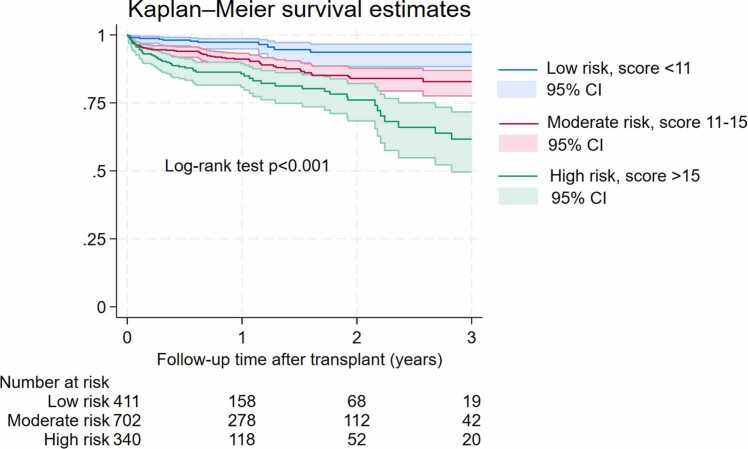
DCD heart transplant survival stratified by risk categories. Patients were grouped into low risk (<11), moderate risk (11−15), and high risk (>15) based on cumulative risk scores. The high-risk group demonstrated significantly reduced survival compared to low- and moderate-risk groups (p<0.001), supporting the prognostic utility of the risk score.

A simplified risk score model was developed using the 14 most significant predictors from multivariable analysis. Each factor was assigned a score based on its regression coefficient, which was its relative contribution to the overall mortality risk. Among donor characteristics, diabetes or male sex received the highest score (4 point each), while CMV positivity (in either donor or recipient) was assigned 3 points. Donor death of head trauma, ischemic time >4 h and lung retrieval were assigned 2 point each. Donor age >=45 or donor death of stroke received 1 point On the recipient side, the highest score (6 points) was attributed to pre-transplant ventilator use. Next was female sex (4 points). Age >65, diabetes, pre-transplant cardiac surgery, and pre-transplant dialysis each contributed 3 points, while use of IV inotropes added 1 point. Based on cumulative scores, patients were stratified into three risk categories: low risk (<11), moderate risk (11–15), and high risk (>15). Details are presented in Table 4. Kaplan-Meier analysis revealed significantly reduced survival in the high-risk group, validating the utility of the score for clinical risk stratification (Figure 1).

**Table 4:** Multivariable model for the 14 most significant factors and their corresponding risk scores.

Risk factors	Hazard ratio [95% CI]	p-value	Coefficients	Score
Donor factors				

Donor age>=45	1.16 [0.50-2.67]	0.732	0.146	1
Male	1.72 [0.96-3.07]	0.067	0.541	4
CMV Donor (+) or CMV Recipient (+)	1.55 [0.97-2.47]	0.068	0.437	3
Diabetes	1.92 [0.83-4.45]	0.127	0.654	4
Ischemic time >4 hours	1.33 [0.91-1.96]	0.144	0.288	2
Donor cause of death				
Anoxia	Ref			
Stroke Head trauma	1.22 [0.60-2.50] 1.29 [0.90-1.84]	0.587 0.162	0.199 0.254	1 2
Lung retrieval	1.33 [0.90-1.96]	0.151	0.285	2

Recipient factors				

Age>65	1.45 [0.98-2.16]	0.064	0.374	3
Female	1.73 [1.13-2.66]	0.012	0.550	4
Diabetes	1.56 [1.10-2.22]	0.012	0.447	3
Pre-transplant dialysis	1.63 [0.78-3.41]	0.192	0.491	3
Pre-transplant cardiac surgery	1.53 [1.08-2.17]	0.018	0.425	3
Pre-transplant IV inotropes	1.23 [0.85-1.78]	0.269	0.208	1
Pre-transplant on ventilator	2.46 [0.60-10.15]	0.213	0.900	6

Low risk: Total score <11; Moderate risk: Total score 11-15; High risk: Total score >15

## Discussion

Due to the shortage of the donor pool, there has been an increasing interest in using DCD hearts in the US since 2019. Most studies showed similar survival between DCD and DBD heart recipients within the first two years after transplant.^9^, ^10^, ^11^, ^12^, ^13^ But recent studies showed potential worse survival in DCD group from year 3 post-transplant.7., 14 It raises the question of what the mortality risk factors in DCD heart transplants are. There have been heart transplant mortality risk scores developed from UNOS data considering the characteristics of donors^21^, ^26^, recipients^16^ or both^15^, ^17^, ^18^, ^19^, ^20^, ^22^. However, the training data were before the start of DCD heart transplantation in the US in 2019. Therefore, these risk scores might not be applicable to predict the mortality risk of DCD heart recipients. By utilizing UNOS data from 2019 to 2024, we had the updated data to assess the DCD mortality risk factors from both donors’ and recipients’ perspectives in the US and developed the corresponding risk scores. To the best of our knowledge, there has not been a published risk score for DCD heart transplant survival.

In our study, we found the following statistically significant risk factors associated with increased mortality in DCD heart recipients: recipient age >65, female sex, diabetes, prior cardiac surgery; donor male sex, prolonged ischemic time, and CMV mismatch. Some of our risk factors such as recipient older age, female sex, diabetes, prior cardiac surgery, and longer ischemic time were also included in published risk scores for DBD heart transplant survival.^15^, ^16^, ^17^, ^18^, ^19^, ^20^, ^21^, ^26^ Other DBD heart transplant risk scores included older donor age and recipient pre-transplant condition such as: dialysis, IV inotropes and mechanical ventilator. We also found these factors associated with an increased mortality in DCD heart transplants but not statistically significant. However, they were kept in the final model given their large effect sizes and clinical relevance. If we excluded other clinically relevant variables with large effect sizes and kept only statistically significant variables then there were only 3 variables left in the model: recipient sex (female HR=1.51, p=0.035), recipient diabetes (HR=1.47, p=0.026) and recipient history of cardiac surgery (HR=1.56, p=0.010). The optimism-corrected C-statistic of this parsimonious model is only 0.60 (95% CI 0.55–0.64) and we believe it is hard to persuade clinicians to use an over-simplified score from this model to predict their patient survival.

We identified CMV mismatch as significant risk factors not mentioned in other risk scores. CMV mismatch is a well-established risk factor for CMV disease, which might lead to graft rejection and cardiac allograft vasculopathy.^27^, ^28^ However, recent studies showed no statistically significant effect of CMV mismatch on heart transplant survival.^29^, ^30^, ^31^ It might be due to the protective effect of antiviral prophylaxis and treatment. There is no direct published evidence comparing the impact of CMV mismatch on mortality specifically stratified for DCD and DBD heart transplants. However, we could hypothesize that DCD hearts may experience longer ischemic times and require normothermic regional perfusion or ex vivo perfusion, which could exacerbate endothelial injury by CMV, the mechanism of cardiac allograft vasculopathy.32., 33 Further studies are needed to evaluate the effect of CMV infections on DCD heart recipients to recommend CMV screening and treatment strategies specifically for this patient population.

Interestingly, we found male donor significantly associated with increased mortality in DCD heart recipients after adjusting for recipient sex, donor/recipient weight ratio and other relevant covariates as mentioned in Table 3. In DBD heart transplants, on the contrary, female donor has been found to be associated with higher mortality in other studies.^17^, ^34^, ^35^ The difference could be due to confounding effects that were not completely controlled in these studies as well as ours. Donor male sex might just be a surrogate factor that could be associated with other comorbidities that might have led to higher survival rates observed in our DCD heart recipients. On the other hand, DCD hearts often have longer ischemic time and require resuscitation as compared to DBD hearts. Therefore, we could hypothesize that male donor hearts may be more susceptible to ischemia-reperfusion injury possibly due to differences in mitochondrial function, oxidative stress response, or hormonal modulation.^36^, ^37^ Estrogen has been shown to exert protective effects on the heart by modulating adrenergic signaling, reducing oxidative stress, and influencing vascular tone while testosterone may enhance sympathetic activity and catecholamine responsiveness, contributing to higher stress reactivity and cardiovascular risk in males.^38^ In addition, studies have shown DCD hearts had more profound changes in myocardial edema, inflammation, and injury than DBD hearts^39^, ^40^ but whether this effect is more prominent in males is to be investigated.

### Study limitations

The UNOS database lacks certain key variables that could influence transplant outcomes, such as the interval between withdrawal of life support and circulatory arrest, as well as the duration from asystole to reperfusion. For DCD hearts maintained on ex vivo perfusion systems without experiencing actual ischemia, the true ischemic time remains unclear. Moreover, the registry does not document the specific procurement techniques used for DCD hearts - whether normothermic regional perfusion (Abdominal NRP or Thoracoabdominal NRP) or direct procurement followed by ex vivo perfusion. Therefore, we might not account for unmeasured confounders in our model. In this context, we used a total ischemic time cutoff of 4 h because it represents the current evidence‑based standard recommended by the International Society for Heart and Lung Transplantation (ISHLT). The 2023 ISHLT donor heart selection guideline specifically advises targeting a total ischemic time ≤4 h to reduce the risk of primary graft dysfunction and early mortality.^41^ Given that UNOS does not provide warm ischemia metrics or procurement‑strategy–specific data (e.g., NRP vs ex‑vivo perfusion), total ischemic time is the only universally available and standardized ischemia parameter across centers and eras. Applying the ISHLT‑endorsed 4–hour threshold therefore ensures consistency with established clinical practice and allows our findings to be compared meaningfully with prior studies, even though it may not fully capture the heterogeneity of contemporary DCD procurement strategies.^17^, ^18^, ^19^, ^20^, ^34^, ^35^

Our study focused exclusively on DCD heart transplantation, a practice that was only initiated in the United States in 2019. Consequently, the available study population was relatively small, with shorter follow‑up duration compared with the more established DBD heart transplant cohort in the UNOS database. To preserve model power, we performed internal validation using 1000–replication bootstrapping rather than splitting the dataset into separate training and testing cohorts. As the national volume of DCD heart transplantation continues to increase, future external validation using larger and more mature datasets will be important. The modest sample size of contemporary DCD recipients also contributed to wider confidence intervals for some risk‑score categories in Figure 1; however, we anticipate that the precision of these estimates will improve as additional DCD transplant data become available. Therefore, the present risk score should be interpreted as exploratory and hypothesis‑generating rather than adoptable for clinical decision‑making. With only 14 risk factors, our model still had a good discrimination power with an optimism-corrected C-statistics of 0.71 (95% CI 0.67- 0.75) which is equivalent or slightly higher than other risk score models.^17^, ^18^, ^19^, ^20^, ^34^, ^35^. As contemporary DCD transplant volume grows, and more granular procurement‑related data become available, future studies will be essential to externally validate and refine this model before clinical implementation.

## Conclusions

Using UNOS data, we successfully developed a mortality risk score for DCD heart transplants including 7 recipient factors: age >65, female sex, diabetes, prior cardiac surgery, pretransplant ventilator use, IV inotropes; 7 donor factors: age≥45, male sex, diabetes, ischemic time >4 h, donor causes of death, lung retrieval and CMV positive in either donor or recipient. It is the initial work that needs further validation.

## FINANCIAL DISCLOSURE STATEMENT

All authors declare that they have no known competing financial interests or personal relationships that could have appeared to influence the work reported in this paper. This project does not have funding support from any sources.

## Declaration of Competing Interest

The authors declare that they have no known competing financial interests or personal relationships that could have appeared to influence the work reported in this paper.
